# A Conversation with Belén González-Gaya

**DOI:** 10.1021/acscentsci.5c01511

**Published:** 2025-08-27

**Authors:** XiaoZhi Lim

## Abstract

The environmental scientist hitched a ride on a tourist cruise to measure pollutants in Antarctica.

## Graphical abstract

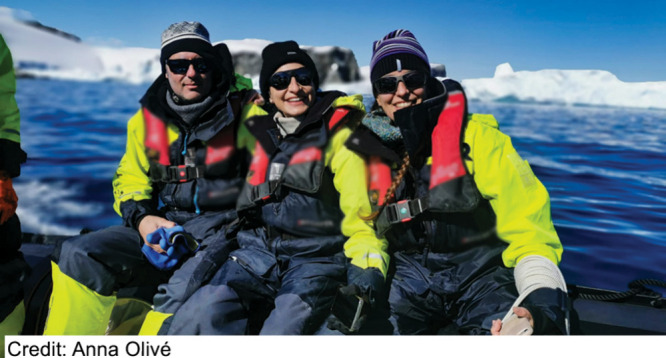

Belén González-Gaya caught the travel bug early in her career. During her PhD studies, the environmental scientist, now at the University of the Basque Country, spent 3 months on board a research cruise that circumnavigated the globe, measuring environmental pollutants in the oceans. González-Gaya has been hunting contaminants in remote regions ever since.

**Figure:**
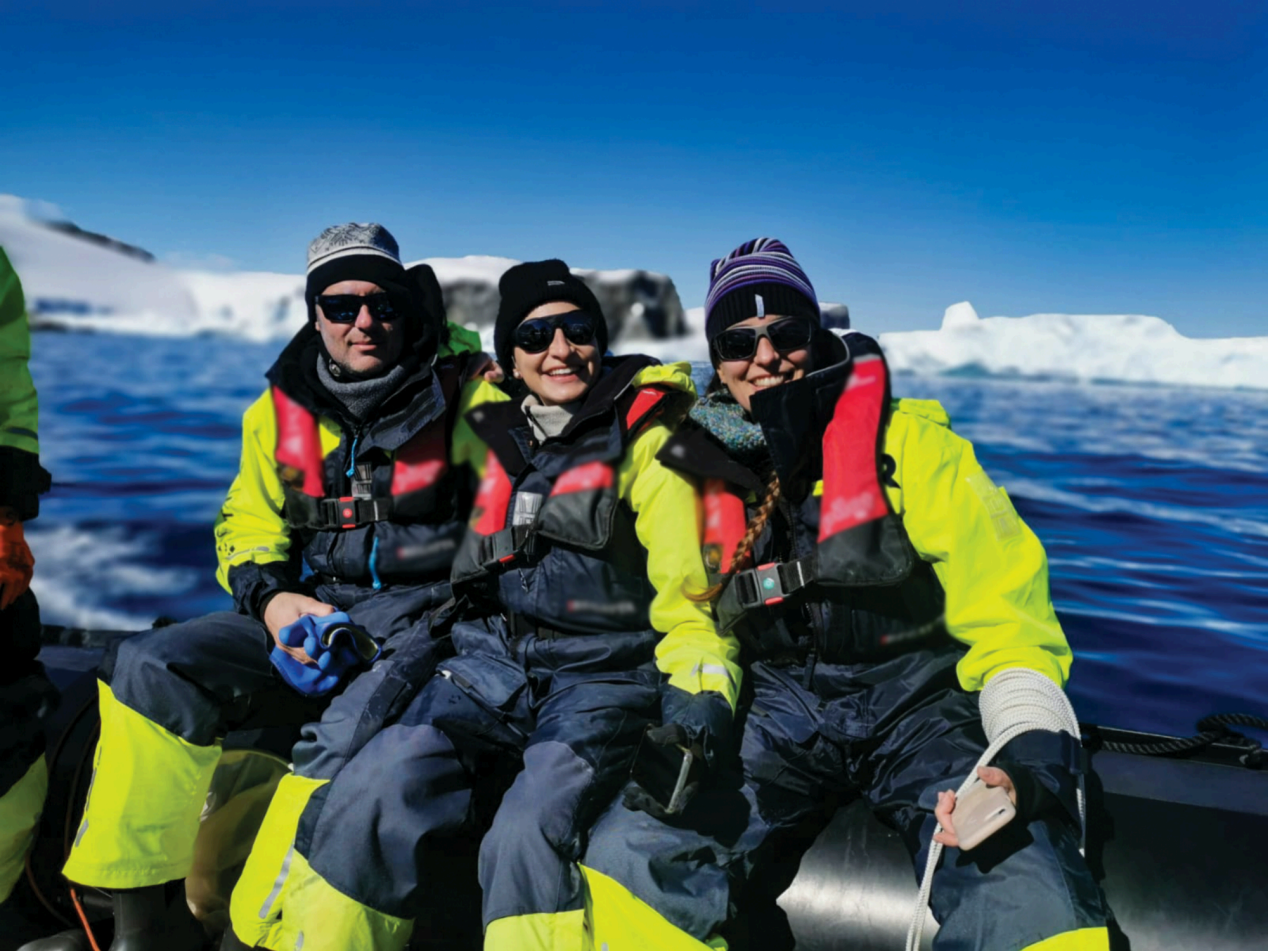
Johan Etourneau (left), Paula Fragueiro Sabaini (middle), and Belén González-Gaya sit on an inflatable boat for collecting plankton and seawater samples near Trinity Island, western Antarctic Peninsula. Credit: Anna Olivé.

Over the years, she grew more interested in contaminants of emerging concern, which, unlike traditionally studied organic pollutants, don’t last as long or travel as far. Yet they can still be detected in remote regions due to human activities there.

On her destination list was the far-flung continent of Antarctica. But despite it having over 60 research bases, getting there as a scientist isn’t easy. “You need to apply for time in the bases, you need to apply for time in the boat,” she says. Plus, projects there are usually led by more well-established researchers, she says.

Still, plenty of people visit Antarctica; they just aren’t researchers. Some 540 voyages carried more than 118,000 tourists there in the southern summer that extended from 2024 to 2025. So in 2023, when a colleague told González-Gaya about an unusual arrangement he had experienced—to join a commercial cruise as a working scientist for tourists to observe and interact with—she jumped at the opportunity.

XiaoZhi Lim spoke to González-Gaya about her trip and the potential for studying remote regions such as Antarctica through tourist travel. This interview was edited for length and clarity.

## What draws you to Antarctica?

I’ve always been fascinated by remote areas. I really like sailing, I love the ocean, I love traveling. I travel a lot.

And when I started measuring pollutants, I felt like, oh my god, they are arriving everywhere. I had the chance to go to many other places—I have samples from the Amazon, from many parts in Asia. But still I was missing the poles. I felt like, come on, I should have gone to Antarctica at some point.

## How did you carry out research on a tourist cruise?

I was very lucky because the company—the boat I was traveling in—is really well prepared. They have two full laboratories: a wet laboratory where you can [handle] your dirty samples, your filtration, the snow, or whatever biological or environmental sample you get. And they have also a dry lab, which is more like a bench lab, where you can do proper biochemistry or chemical analysis.

## What was it like to share your work with the tourists?

You go there for free, you get a lot of space, but at the same time, you are presented to the tourist as a scientist. You are not a worker of the company, but some outreach was part of the deal. You need to give talks. You need to be available if any tourists want to talk to you or visit your lab or know more about your research.

It’s a challenge, but at the same time, it’s an opportunity because otherwise [the tourists] would never go to a talk I would be offering.

## What samples did you bring home?

Between 100 and 150 water samples, 50 snow samples, and then we’ve also got plankton.

We are not allowed to take liters and liters of Antarctic water to Spain, so for instance, for the water or the snow samples, we did solid-phase extraction. So you just take your filtering systems, concentrate all the pollutants on a sorbent, and then we take the sorbent [back to Spain].

**Figure:**
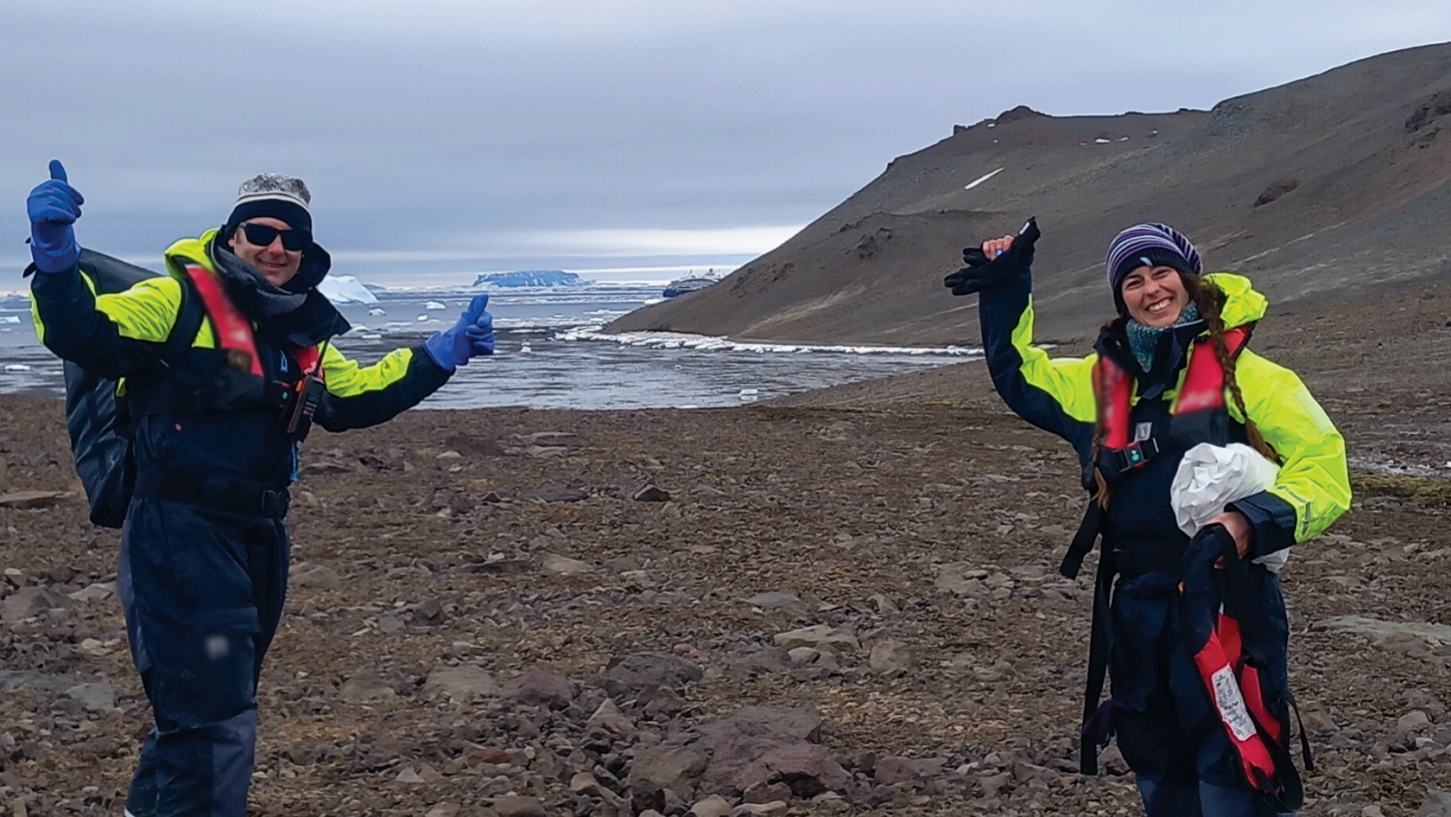
Johan Etourneau (left) and Belén González-Gaya walk to get snow samples on Vega Island, eastern Antarctic Peninsula. Credit: Paula Fragueiro Sabaini.

## What contaminants did you target in the samples?

We are focused on contaminants of emerging concern; those would not necessarily be new chemicals but substances that we are not really aware are pollutants. [For example,] caffeine, aspirin, personal care products—so the UV [ultraviolet] filters that you can find in sunscreen. Also we measure many endocrine disruptors such as plasticizers, maybe the bisphenols or parabens that you can find in some creams.

Many pharmaceuticals, really common things that we use—they are safe at certain quantities, but when you place them into places where they shouldn’t be or at concentrations much higher than those recommended, they may impact ecosystems.

## How does tourist activity impact Antarctica, chemically speaking?

[Picture] a little island with a penguin colony in the middle. There are a few boats every day arriving there. Some [people] can even step off and go to the penguins to take pictures. The boats that are on the coast at that time, maybe they are [discharging] their water there.

Somehow you don’t expect to find pharmaceuticals, but we confirmed that [they are] in the snow and in the coastal waters. One of the degradation products of benzophenone was fairly abundant, which is normal because you have huge UV exposure in Antarctica, so everyone is using [sun] creams. People do smoke there, [so we also found] not nicotine itself but cotinine, which is a human metabolite. So it’s probably not [from cigarette butt litter] but pee, coming directly through the water.

We are analyzing the plankton at the moment. The plankton would be the first step to figure out if there is some bioaccumulation or some entrance of these emerging contaminants into the trophic chain.

## Should more scientists partner with tourism companies to access remote regions?

I think it’s a very good [opportunity]. But still, I wouldn’t go there with [just] anyone. I would always make sure the [cruise operators] you are collaborating with are fair. You need to know what they have done in the past or why they want to invite you. Is it because they are really interested in research, or is it for selling you as a touristic product, because that could happen too.

I see it the same way as if it was a [research] funding company. Would you work with, I don’t know, Coca-Cola if you’re working with plastics? Maybe [that research would do] something good, but maybe it’s only greenwashing. It is a really deep ethics issue that we need to talk about.

When I was in Antarctica, there was a researcher from Australia working in whale watching. He’s gone there for five, six years. He had traveled with many boats, with many companies. He says that some are better than others. But in general, he told me that he was taking this opportunity.

## What else could the scientific community do for remote regions like Antarctica?

I think that the international community and research scientific community should push for enforcing laws in these [polar] areas. There are no governments in Antarctica or in the Arctic or in these international waters, but I do think that when there is tourism, when there are human activities, those should be regulated.

Antarctica is really becoming chaotic. The last season in the Antarctic Peninsula has been really overcrowded. So we should do something about that. We should give it a thought.

*XiaoZhi Lim is a freelance contributor to* Chemical & Engineering News, *the independent news outlet of the American Chemical Society.*

